# Perceived Environmental and Personal Factors Associated with Walking and Cycling for Transportation in Taiwanese Adults

**DOI:** 10.3390/ijerph120202105

**Published:** 2015-02-13

**Authors:** Yung Liao, I-Ting Wang, Hsiu-Hua Hsu, Shao-Hsi Chang

**Affiliations:** 1 Department of Health Promotion and Health Education, National Taiwan Normal University, 162, Heping East Road Section 1, Taipei 10610, Taiwan; E-Mails: liaoyung@ntnu.edu.tw (Y.L.); a5210701@hotmail.com (I.-T.W.); 2 Department of Physical Education, National Taiwan Normal University, 162, Heping East Road Section 1, Taipei 10610, Taiwan; E-Mail: t08016@ntnu.edu.tw

**Keywords:** perceived environment, active commuting, Taiwanese, walking for transportation, cycling for transportation, personal factors

## Abstract

This study examined perceived environmental and personal factors associated with walking and cycling as means of transportation for Taiwanese adults. A random-digit-dialing telephone-based cross-sectional survey was conducted with Taiwanese adults aged 20 to 64 years. Data on time spent walking and cycling for transportation and perceptions of neighborhood environment and personal characteristics were obtained from 1065 adults by using the International Physical Activity Questionnaire-long version and its environmental module. Adjusted binary logistic regression was performed. The results showed that, after adjusting potential confounders, common and different personal and perceived environmental factors were associated with walking and cycling for transportation. For common personal factors, adults who had employment were less likely to engage in 150 min of walking per week (odds ratio [OR] = 0.41; 95% confidence interval (CI): 0.27–0.62) and to use cycling as a means of transportation (OR = 0.51; 95% CI: 0.32–0.79). For common perceived environmental factors, adults who perceived good connectivity of streets were more likely to walk (OR = 1.95; 95% CI: 1.20–3.16) and cycle (OR = 2.02; 95% CI: 1.16–3.54) for transportation. Targeting employed adults and improving the connectivity of streets should be a priority for developing transport policies and intervention strategies to promote active transportation.

## 1. Introduction

A lack of physical activity is a major risk factor of obesity and noncommunicable diseases such as cardiovascular disease, type 2 diabetes, and certain types of cancer [1,2]. Despite these adverse consequences, an international study examining the prevalence of physical activity in 20 countries across Asia, Europe, and America indicated that 42.3% of Taiwanese adults failed to achieve the minimum recommendations of physical activity (150 min/week) recommended by the World Health Organization (WHO) [2,3,4] primarily because of a lack of time in daily life [5]. Moreover, the prevalence of overweight (body mass index, BMI ≥ 24 kg/m^2^) in Taiwanese adults was estimated at 38.3% in 2013 [6]. Therefore, there is an urgent need to develop effective strategies to encourage Taiwanese adults to engage in sufficient physical activity that will assist in disease prevention. 

Active transportation by walking and cycling is considered a favorable strategy for encouraging adults to sustain habitual physical activity [7,8]. Furthermore, walking and cycling for transportation was found to be associated with decreased risks of cardiometabolic health and all-cause mortality [9,10,11], as well as the environmental benefits of replacing vehicle use with active movement [12] and increasing environmental protection [13,14]. Like most high- and middle-income countries, private vehicle use including cars and motorcycles, has become the main mode of transportation in Taiwan, with a particularly high rate of car and motorcycle ownership (267.1 per 1000 people for cars; 608.5 per 1000 people for motorcycles) [15]. According to a 2013 report from the Ministry of Transportation and Communication of Taiwan [16], 72.4% of Taiwanese adults used private motorized vehicles as their main mode of daily transportation; only 12.4% of adults walked or cycled. The prevalence of active transportation through walking or cycling was lower when compared with other high- and middle-income countries [17,18,19]. Therefore, to promote active transportation, it is critical to identify associated factors of walking and cycling for transportation in Taiwanese adults. 

From the perspective of the ecological model of health behavior, a deeper understanding of personal and environmental factors related to active transportation is crucial for targeting the population who are most in need of intervention, and to develop effective environmental approaches to provide a long-term impact on active commuting behaviors [20]. Moreover, to develop a tailored intervention strategy for promoting active transportation to targeted groups, the importance of examining specific environmental factors related to context-specific behaviors has been emphasized by Giles-Corti *et al.* [21]. In this context, an increasing number of studies have shown that environmental attributes, whether perceived or objectively measured, play crucial roles in specific transport-related behaviors [19,22,23,24,25,26]. Two previous reviews concluded that objectively-measured environmental attributes, such as higher population density, greater connectivity, and increased mixed land use, were consistently related to an increased amount of time spent walking and cycling for transportation [22]; the objectively-measured presence of cycling paths, short trip distances, proximity of cycling paths, and general traffic dangers were associated with cycling for transportation [23]. However, mixed findings have been reported among countries regarding the association between perceived environment and walking and cycling for transportation [19,24,25]. Therefore, it is crucial to understand how perceptions of environment are related to the use of transportation through walking and cycling in different settings because recent studies have reported that perceived rather than objective environment is a stronger predictor of specific physical activity behavior [27,28]. Moreover, few studies have compared the personal and perceived environmental correlates of transport-related physical activity behavior, particularly in the Asian countries, which likely have different residential densities, culture, and infrastructure than Western countries. Thus, this study examined the perceived environmental and personal factors associated with walking and cycling for transportation purposes for Taiwanese adults.

## 2. Methods 

### 2.1. Respondents

This study used cross-sectional survey data from a random-digit-dialing telephone-based survey conducted from May to June 2014 through a telephone research service company in Taiwan. In May 2014, Taiwan was estimated to have a population of 23,386,883 and an area of 36,192.8 km^2^. A total of 67.1% of inhabitants were the target population for this study (adults aged 20–64 years; *N* = 15,782,252). The required sample size for this study was calculated to be 1067 adults with a 95% confidence level and a 3% confidence interval. A stratified and clustered multistage sampling process was used to select respondents. Trained interviewers administered a standardized questionnaire. All the interviewers had experience in administering telephone population surveys and received 2 days of training before the start of each survey. A total of 1528 adults were asked, and 1083 of them completed the survey (response rate: 70.9%); however, after data cleaning, only 1065 participants submitted valid data for analysis. Each telephone interview was not longer than 20 min to ensure validity. The telephone research service company did not offer any rewards for participation. Verbal informed consent was obtained before the start of the telephone interviews and the study protocols were reviewed and approved by the Ethics Committee of National Taiwan University.

### 2.2. Measures

#### 2.2.1. Outcome Variables 

The outcome variables of walking and cycling for transportation were obtained from the Taiwanese version of the International Physical Activity Questionnaire-long-version (IPAQ-LV) [29], which has been widely used in self-administrated [25,30,31] and telephone-based surveys [19,32]. Because the distribution of time spent on walking and cycling for transportation was skewed, the outcome variables were dichotomized on the basis of the recommendation of the IPAQ scoring protocol [33]. Walking for transportation was categorized according to the recommendation for physical activity and health (satisfying 150 min/week) by the WHO [2,34]. Bicycling for transportation was divided into two categories, namely yes and no, on the basis of the median (0 min/week).

#### 2.2.2. Perceived Environmental Variables 

Perceived environmental factors were measured with the Taiwanese version of the International Physical Activity Questionnaire-environmental module (IPAQ-E). The IPAQ-E questionnaire was developed by the International Physical Activity Prevalence Study to understand the environmental factors affecting walking and bicycling in neighborhoods, and was used in several countries [35,36,37]. The IPAQ-E was translated with the IPAQ [29] according to the process of translation and adaptation of instruments from the WHO [38]. A pretest was administered to 111 adults who were not part of the main study. Pretest respondents were asked if there were any words they did not understand, and if they found any words or expressions unacceptable or offensive. A retest of the questionnaire was conducted 1 week later. The IPAQ-E questionnaire consisted of three categories of items, which included seven core items, four recommended items, and six optional items [39]. These 17 items were measured using a 4-point Likert scale (*strongly agree*, *somewhat* *agree*, *somewhat* *disagree*, and *strongly* *disagree*), except for two questions: First, “what is the main type of housing in your neighborhood?” For this question, the five options were detached single-family housing, apartments with two to three stories, mix of single-family housing and apartments with two to three stories, condos with four to 12 stories, and condos with greater than or equal to 13 stories. Second, “how many cars or motor bikes are there in your household?” This question was open ended [39]. In this study, 12 of the 17 items were included for measuring the perceived environmental attributes: (1) residential density, (2) access to shops (*Many shops are within walking distance of my home*), (3) access to public transport *(It is less than a 10–15 min walk to a transit* *station* *from my home)*, (4) presence of sidewalks *(There are sidewalks on most of the streets in my* *neighborhood)*, (5) presence of bike lanes *(There are facilities to cycle in or near my neighborhood)*, (6) access to recreational facilities *(My neighborhood has several free or low cost recreation facilities)*, (7) crime safety at night *(The crime rate in my neighborhood makes it unsafe to go on walks at night)*, (8) traffic safety *(There is so much traffic on the streets that walking is difficult or unpleasant)*, (9) seeing people being active *(I see many people being physically active in my neighborhood)*, (10) aesthetics *(There are many interesting things to look at while walking in my neighborhood)*, (11) connectivity of streets (*There are many four-way intersections in my neighborhood)*, and (12) presence of destination *(There are many places to go within easy walking distance of my home)*. Four optional items regarding the traffic safety for bicyclists, maintenance of sidewalks, maintenance of bike lanes, and safety from crime during the day were not included in this study. The item “having cars or motor bikes” was categorized as a personal variable. Detailed descriptions of these items are available online (http://www.drjamessallis.sdsu.edu/ measures.html). Similar to the processes in previous studies [37,40], these 12 environmental variables were converted into binary items. Residential density was divided into “detached single-family housing” and “others”. The other 11 items were categorized as “agree” *(strongly* *agree* and *somewhat* *agree)* and “disagree” *(somewhat* *disagree* and *strongly* *disagree)*.

#### 2.2.3. Personal Variables 

Personal factors included gender, age, residential area, educational level, occupational type, marital status, living status, and BMI. Age was divided into four categories: 20–29 years, 30–39 years, 40–49 years, and 50–64 years. Residential area was categorized into “metropolitan” and “nonmetropolitan” areas. Educational level was classified into two groups: “high school degree or lower” and “university degree or higher”. Occupational type was categorized into “employment” and “not employment” (including retirement, unemployment, and student statuses). Marital status was classified as “married” and “not married” (including widowed, separated, and divorced). Living status was divided into “living with others” and “living alone.” BMI was based on self-reported weight and height and was grouped into two categories: “not overweight” (<24 kg/m^2^), “overweight/obese” (≥24 kg/m^2^). Finally, “having cars or motor bikes” was measured using the item “how many cars or motor bikes are there in your household.” This question was open ended. “Having cars or motor bikes” was divided into “one or more” and “none.” 

### 2.3. Statistical Analyses

The data were analyzed from 1065 Taiwanese adults who provided complete information for the study variables. Forced-entry adjusted logistic regression for gender, age, residential area, educational level, occupational type, marital status, living status, BMI, and vehicle ownership was conducted to examine the association of nine personal and 13 perceived environmental factors for walking and bicycling for transportation purposes, respectively. Adjusted odds ratios (ORs) and 95% CIs were calculated for each variable. Inferential statistics were performed using IBM SPSS 22.0 (IBM Corporation, Armonk, NY, USA), and the level of significance was set at *p* < 0.05.

## 3. Results 

### 3.1. Characteristics of Participants

Table 1 presents the characteristics of the respondents (mean age: 42.7 ± 12.6 years). The sample was well balanced between gender and age. Of the respondents, 60.4% lived in metropolitan areas, 60.5% had a university education or higher degree, 66.4% had full-time employment, 65.2% were married, 95.7% were living with others, 35.4% were overweight or obese, and 97.8% owned cars or motor bikes. 

The prevalence of achieving 150 min/week of walking for transportation purposes was 13.7%. Furthermore, in the last 7 days, the prevalence of walking for transportation purposes was 37.4% and the prevalence of cycling for transportation purposes was 10.9%.

**Table 1:** Basic characteristics of all respondents (*n* = 1065).

Basic Characteristics	Sample of This Study		National Data ^a^
	N	%	%
**Gender**			
Men	540	50.7%	50.0%
Women	525	49.3%	50.0%
**Age (Year)**			
50–64	353	33.1%	31.4%
40–49	263	24.7%	23.1%
30–39	245	23.0%	25.0%
20–29	204	19.2%	20.5%
**Residential area**			
Non-metropolitan	422	39.6%	40.0%
Metropolitan	643	60.4%	60.0%
**Educational level**			
High school degree or lower	421	39.5%	52.5%
University degree or higher	644	60.5%	47.5%
**Occupational type**			--- **^b^**
Employment	707	66.4%	---
Not employment	358	33.6%	---
**Marital status**			
Not married	371	34.8%	33.7%
Married	694	65.2%	66.3%
**Living status**			--- **^b^**
Living alone	46	4.3%	---
Not living alone	1019	95.7%	---
**Body Mass Index (kg/m^2^)**			
Non-overweight	688	64.6%	61.7%
Overweight/obese	377	35.4%	38.3%
**Household car or motor bikes ownership**			--- **^b^**
One or more	1042	97.8%	---
None	23	2.2%	---
**Walking for transportation**			--- **^b^**
<150 min/week	919	86.3%	---
150+ min/week	146	13.7%	---
**Walking for transportation**			--- **^b^**
No (0 min/week)	667	62.6%	---
Yes	398	37.4%	---
**Cycling for transportation**			--- **^b^**
No (0 min/week)	949	89.1%	---
Yes	116	10.9%	---

**^a^** Data source: [6,15,56]; **^b^** ---: National data could not be found to compare with our data.

### 3.2. Personal Factors Associated with Walking for Transportation 

The ORs for attaining 150 min/week of walking and cycling are presented in Table 2 according to gender, age, residential area, educational level, occupational type, marital status, living status, BMI, and car or auto bike ownership. Table 2 shows that adults who lived in nonmetropolitan areas (OR = 0.42; 95% CI: 0.28–0.65), had employment (OR = 0.41; 95% CI: 0.27–0.26), and had one or more cars or motor bikes (OR = 0.22; 95% CI: 0.09–0.57) were less likely to achieve 150 min or more of walking as a method of transportation. 

### 3.3. Personal Factors Associated with Cycling for Transportation 

Table 2 also shows that respondents aged 20 to 29 years (OR = 0.46; 95% CI: 0.21–0.99), who had employment (OR = 0.51; 95% CI: 0.32–0.79), and who were married (OR = 0.50; 95% CI: 0.29–0.85) were less likely to engage in cycling as a method of transportation. 

**Table 2:** Personal factors associated with walking and cycling for transportation.

Personal Factors	Walking for Transportation	Cycling for Transportation
	OR (95% CI)	OR (95% CI)
**Gender**		
Men	1.00	1.00
Women	0.88 (0.58–1.33)	0.90 (0.58–1.40)
**Age**		
50–65	1.00	1.00
40–49	1.15 (0.70–1.87)	0.68 (0.41–1.45)
30–39	0.72 (0.40–1.32)	0.77 (0.38–1.21)
20–29	0.55 (0.26–1.15)	0.46 (0.21–0.99) *
**Residential area**		
Metropolitan	1.00	1.00
Non-metropolitan	0.42 (0.28–0.64) **	0.83 (0.55–1.26)
**Educational level**		
High school or lower	1.00	1.00
University or higher	1.07 (0.72–1.60)	0.96 (0.62–1.49)
**Occupational type**		
Not employment	1.00	1.00
Employment	0.41 (0.27–0.62) **	0.51 (0.32–0.79) **
**Marital status**		
Not married	1.00	1.00
Married	0.76 (0.45–1.28)	0.50 (0.29–0.85) *
**Living status**		
Living alone	1.00	1.00
Not living alone	2.12 (0.66–6.75)	1.84 (0.60–5.66)
**Body Mass Index status**		
Non-overweight	1.00	1.00
Overweight/obese	1.23 (0.81–1.87)	0.96 (0.60–1.53)
**Household car/motor bikes**		
None	1.00	1.00
One or more	0.25 (0.10–0.63) *	0.40 (0.15–1.05)

Adjusted for gender, age, residential area, educational level, occupational type, marital status, living status, BMI status, vehicle ownership; * *p* < 0.05, ** *p* < 0.001.

### 3.4. Perceived Environmental Factors Associated with Walking for Transportation

Logistic regression analyses revealed that six of 13 environmental attributes were significantly associated with 150 min/week of walking for transportation purposes (Table 3). Respondents who perceived good access to public transport (OR = 1.71; 95% CI: 1.00–2.93), the presence of sidewalks (OR = 1.81; 95% CI: 1.21–2.70), the good connectivity of streets (OR = 1.95; 95% CI: 1.20–3.16), and the presence of destination (OR = 1.95; 95% CI: 1.20–3.16) were more likely to walk 150 min/week or more as a method of transportation. 

**Table 3:** Perceived environmental factors associated with walking and cycling for transportation.

Perceived Environmental Factors	N	%	Walking for Transportation	Cycling for Transportation
			OR (95% CI)	OR (95% CI)
**Residential** **density**				
High	954	89.6%	1.40 (0.73–2.69)	1.60 (0.76–3.35)
Low	111	10.4%	1.00	1.00
**Access to shops**				
Good	1012	95.0%	3.89 (0.92–16.40)	0.67 (0.28–1.60)
Poor	53	5.0%	1.00	1.00
**Access to public transport**				
Good	865	81.2%	1.71 (1.00–2.93) *	1.52 (0.85–2.70)
Poor	200	18.8%	1.00	1.00
**Presence of sidewalks**				
Yes	651	61.1%	1.81 (1.21–2.70) *	1.28 (0.83–1.97)
No	414	38.9%	1.00	1.00
**Presence of bike lanes**				
Yes	349	32.8%	1.32 (0.90–1.92)	1.63 (1.08–2.46) *
No	716	67.2%	1.00	1.00
**Access to recreational facilities**				
Good	892	83.8%	1.61 (0.93–2.80)	1.08 (1.60–1.89)
Poor	173	16.2%	1.00	1.00
**Crime safety** **at** **night**				
Not safe	198	18.6%	0.93 (0.58–1.47)	0.73 (0.44–1.21)
Safe	867	81.4%	1.00	1.00
**Traffic safety**				
Not safe	400	37.6%	0.79 (0.55–1.14)	0.99 (0.65–1.52)
Safe	665	62.4%	1.00	1.00
**Seeing people being active**				
Yes	733	68.8%	0.98 (0.66–1.45)	1.07 (0.68–1.69)
No	332	31.2%	1.00	1.00
**Aesthetics**				
Yes	539	50.6%	1.25 (0.86–1.80)	1.95 (1.27–3.00) *
No	526	49.4%	1.00	1.00
**Connectivity of streets**				
Yes	793	74.5%	1.95 (1.20–3.16) *	2.02 (1.16–3.54) *
No	272	25.5%	1.00	1.00
**Presence of destination**				
Yes	749	70.3%	1.91 (1.21–3.02) *	1.50 (0.93–2.42)
No	316	29.7%	1.00	1.00

Adjusted for gender, age, residential area, educational level, occupational type, marital status, living status, BMI, vehicle ownership; * *p* < 0.05.

### 3.5. Perceived Environmental Factors Associated with Cycling for Transportation 

Table 3 shows that adults who perceived presence of bike lanes (OR = 1.63; 95% CI: 1.08–2.46), good aesthetics (OR = 1.95; 95% CI: 1.27–3.00), and good connectivity of streets (OR = 2.02; 95% CI: 1.16–3.54) were more likely to use cycling for transportation. 

## 4. Discussion

This is one of the few studies from Asian countries that examines the associations of personal and perceived environmental factors with walking and cycling for transportation in Taiwanese adults. The most crucial findings of the present study are that, after controlling for sociodemographic covariates, a common personal factor, employment (negatively), and a common perceived environmental factor, the connectivity of streets (positively), are both related to cycling and walking as a means of transportation for 150 min/week. Moreover, because the importance of examining specific environmental factors relating to context-specific behaviors was emphasized [21], perceived environmental attributes were found to be associated with different context-specific physical activity behaviors in previous studies [19,20,25,32,41,42]. The results of the present study support the findings of different perceived environmental factors and reveal that different personal characteristics were associated with walking for transportation and cycling for transportation. Therefore, these results may have crucial implications for policy makers or intervention designers when developing common and different intervention strategies to promote walking and cycling as methods of transportation. 

Street connectivity, the directness or ease of travel between two points that is directly related to the characteristics of street design, has been consistently found to encourage people to walk or cycle [22,43], with the same results found in this study. Therefore, increasing street connectivity should be a priority when designing walkable and bikable neighborhood environments. In addition to street connectivity, another critical finding of the present study is that four perceived environmental factors (*i.e.*, access to public transportation, presence of sidewalks, traffic safety for bicyclists, and the presence of destination) were associated with 150 min of walking as a means of transportation, whereas two perceived environmental attributes (*i.e.*, the presence of bike lanes and aesthetics) were related to having cycled as a means of transportation in the past 7 days. Walking for transportation purposes, and consistent with previous review articles [43,44], the presence of destination, good access to public transportation, and the presence of sidewalks were positively related to walking as a means of transportation. A possible reason is that neighborhood environments with good access to local shops, services, and public transportation stops through sidewalks and better street connectivity may motivate adults to walk more when going places [43]. For cycling as a means of transportation, the present result showed that aesthetics was positively related, which is consistent with results reported in the United States, Belgium, and Australia [25]. In addition, and consistent with a previous study [23] and review [45], a positive association between the presence of bike lanes and cycling as a means of transport was also observed. These results may strengthen the evidence for several perceived environmental attributes associated with walking or cycling across countries with various cultures and environments, which is crucial for the literature because it has a limited amount of data reported from Asia countries. 

The results of this study also indicated that common and different personal factors were associated with walking and cycling for transportation purposes. Previous studies have reported that adults who were male and young were more likely to cycle as a means of transportation [17,19,46,47,48], whereas adults with low educational levels were more likely to walk [19,22,44,46]. However, limited studies have addressed the relationship between job status and walking or cycling. The present results showed that employment is negatively associated with walking and cycling for transportation purposes. This could be because adults with employment may be required to travel from the home to the workplace, which is a longer travelling distance (non-neighborhood setting) compared with those without employment who may be more likely to have local destinations in the neighborhood [43]. Thus, employed adults may be less likely to use the mode of walking or cycling for transportation for long commuting trips. These results may suggest that intervention strategies (e.g., the promotion of public transport use) should target adults with employment, which might be a potential subgroup who has a regular route to their work [49]. Moreover, Taiwanese adults living in nonmetropolitan areas and who have cars or motor bikes were less likely to walk, which is consistent with previous findings [22,24,50]. The possible reasons might be that nonmetropolitan areas may have a lower residential density, less infrastructure, and longer distances to public transportation services or destinations [51]. Adults who have cars or motor bikes may also be more dependent on their private vehicles. Moreover, inconsistent with previous studies [17,19], this study showed that younger adults (20–29 years) and those who were married were less likely to cycle for transportation purposes. A possible explanation for this result could be that younger adults in Taiwan are less physically active and less likely to have regular exercise than older adults [5], which might differ among countries [4,17,19]. Furthermore, although being married has been found to be associated with several positive health behaviors and outcomes [52], adults may receive concern from their families when using cycling as a means of transportation. In contrast with a previous review [9], overweight adults were not found to be less likely to walk or cycle for transportation than were those who were of normal weight. A possible reason for this could be that the preference for transport-related physical activities of Taiwanese overweight adults is different compared to other countries. Additional studies on this topic are warranted. These results implied that walking and cycling are associated with common and different personal factors; thus, these factors must be taken into consideration when designing tailored and effective intervention.

Several limitations to the current study should be considered. First, the study had a cross-sectional design, making it impossible to determine causality. Second, the main measurements, walking and cycling for transportation purposes and environmental factors, were self-reported and could be subject to bias [53,54]. Finally, the study had a limited ability to obtain representative samples because it relied on a telephoned-based survey; therefore, including the parts of the population that did not have a household telephone (approximately 5.3% in 2013) [55] was impossible. Moreover, compared with national data [6,15,56], the respondents of the present study had higher educational levels and a higher prevalence of being overweight. Thus, the results in the present study may be less applicable to the general population. 

## 5. Conclusions 

The results of this study suggest that both common and different personal and environmental strategies should be considered for promoting walking and cycling as a means of transportation. Targeting employed adults and improving the connectivity of streets should be a priority for developing transport policies and intervention strategies to promote active transportation. 
